# Factors Influencing Hospitalization Cost for Diabetic Patients in Traditional Chinese Medicine Hospitals in Qingyang City, China

**DOI:** 10.3390/ijerph192113859

**Published:** 2022-10-25

**Authors:** Mengen Chen, Fanxin Kong, Bin Su, Xuexuan Wei, Jingyu Yang

**Affiliations:** 1 School of Public Health, Gansu University of Chinese Medicine, Lanzhou 730000, China; 2 School of Public Health, Lanzhou University, Lanzhou 730000, China; 3 School of Business and Management, Gansu University of Chinese Medicine, Lanzhou 730000, China

**Keywords:** diabetes mellitus, hospitalization cost, TCM hospitals, Qingyang City

## Abstract

(1) Background: This study aims to understand the factors influencing hospitalization cost related to diabetes mellitus in two traditional Chinese medicine (TCM) hospitals, and to provide a scientific basis for TCM hospitals to control the hospitalization cost of chronic diseases, including diabetes mellitus. (2) Methods: Univariate analysis was used to preliminarily screen the factors related to hospitalization cost, and then multiple linear regression and path models were comprehensively used to analyze the influencing factors of hospitalization cost. (3) Results: The cost of hospitalization for diabetic patients was mainly affected by hospital level, length of stay, type of diabetes, and complications and comorbidities, and hospital level was the most critical influencing factor. (4) Conclusions: The higher the Chinese medicine hospital level, the longer the length of stay, and the more severe the complications and comorbidities, the higher the hospitalization cost for diabetic patients. The Chinese government should continue to promote the tiered medical treatment system and improve the standard of treatment at TCM hospitals to reduce the economic burden of chronic diseases, including diabetes mellitus.

## 1. Introduction

Diabetes mellitus is a chronic disease in which glucose level in the blood exceeds normal level because the body does not produce enough insulin or its biological effects are impaired, which negatively affects a person’s quality of life and survival expectations. It is divided into type 1 diabetes mellitus (T1DM), type 2 diabetes mellitus (T2DM), specific types of diabetes due to other causes, and gestational diabetes mellitus [1,2]. According to the IDF World Diabetes Map (10th Edition) released by the International Diabetes Federation (IDF) in 2021, the number of adults with diabetes in the world reached 537 million in 2021, and the number of adults with diabetes in the world will reach 784 million in 2045. In 2021, there were 141 million adults with diabetes in China, and the number is expected to reach 174 million in 2045 [3]. In addition to this, the prevalence of diabetes in China increased from 10.9% to 12.4% from 2013–2018, and the prevalence has continued to rise in recent years [4]. Thus, diabetes mellitus has become a serious public health problem worldwide, and the problem of diabetes is particularly prominent in China [5].

Existing studies have shown that the annual total hospitalization cost for diabetic patients is higher than that of non-diabetic patients, and the total hospitalization cost of type 1 diabetes mellitus is many times that of type 2 diabetes mellitus [6,7]. In addition, diabetic patients with other comorbidities and complications will consume more medical resources and thus bear more economic burden of the disease. In 2021, the total medical cost for diabetes mellitus and its related diseases in the United States was the highest, reaching about $379.47 billion, while Switzerland has the highest per capita medical cost for diabetes and related diseases, with an average of about $12,828.4, and the top ten countries in diabetes expenditure are mainly concentrated in Europe [3]. In China, the total cost of diabetes and its related diseases is about 16.53 billion dollars, which is only 1173.5 dollars per capita, which is considerably lower than in the developed countries of Europe and America [3]. What’s more, as diabetes rates continue to rise, many countries, including China, will face a heavier economic burden of the disease [6,8,9].

Chinese medicine not only has a long legacy, but also has unique treatment methods, is characterized as being “simple, convenient, experimental, and inexpensive”, and has distinct advantages in treating chronic diseases such as diabetes mellitus [10,11,12]. Therefore, this study retrospectively analyzed the relevant information of inpatients with diabetes in two TCM hospitals that had implemented diagnosis-related group (DRG) reform in Qingyang City, Gansu Province, China from January 2017 to August 2020. These two hospitals were chosen because they represent the standard of medical care in Gansu Province, China, and in the Northwest region. Also discussed are the influencing factors of hospitalization cost of patients with diabetes in TCM hospitals, including countermeasures and suggestions for effective control of hospitalization cost of patients with diabetes in TCM hospitals, and full analysis of the price ratio advantage of treating chronic diseases in TCM hospitals.

## 2. Materials and Methods

### 2.1. Study Design and Population

The data in this study were obtained from the National Health Big Data platform of the Health Commission of Gansu Province, China, from which the medical records of two TCM hospitals in Qingyang City, Gansu Province, which conducted the pilot DRG reform from January 2017 to August 2020, were obtained. According to the International Classification of Diseases, 10th edition (ICD-10), the information of patients with diabetes mellitus, coded as E10–E14, was extracted, and the data for length of stay less than 1 day, logical errors in the information, or incomplete information that could not be effectively supplemented were screened to avoid study bias. Through the above treatment, 1268 effective cases were finally included. The study data should not be made public because they need to be treated confidentially, so please contact the corresponding author if necessary.

### 2.2. Data Processing

To eliminate the biased effect of inflation on the study of hospitalization cost for diabetic patients, the hospitalization cost from 2017 to 2020 were adjusted according to the Consumer Price Index (CPI) of Qingyang City, Gansu Province, China, with 2016 as the base period, so as to conduct statistical correlation analysis.

In this study, the length of stay and hospitalization cost were taken as endogenous variables, and gender, age, visit times, hospital level, surgery and procedures, complications and comorbidities, type of diabetes, and DRG reform were taken as exogenous variables. In addition, according to the actual analysis needs, the classification or integration of relevant variables was completed, and the specific variable assignment processing is shown in Table 1.

### 2.3. Statistical Analysis

The main focus of the paper is on cost assessment from a healthcare perspective and a step-by-step examination of the factors influencing hospitalization cost for people with diabetes mellitus. Firstly, univariate analysis was used to screen the factors related to length of stay and hospitalization cost. Since the original data on the length of stay and hospitalization cost did not follow a normal distribution, the Mann–Whitney U test was used for dichotomic variables, the Kruskal–Wallis H test was used for multiple categorical variables, and the Wilcoxon test was used for the independent variable “DRG reform”. Secondly, statistically significant variables in univariate analysis were selected as independent variables, and the length of stay and hospitalization cost were log-transformed (the length of stay and hospitalization cost after conversion approximately followed a normal distribution). Thus, a multivariate linear regression model with “Ln (length of stay)” and “Ln (hospitalization cost)” as dependent variables could be established. Finally, the statistically significant variables in the results of multiple linear regression were taken as independent variables, the length of stay was taken as the mediating variable, and the hospitalization cost was taken as the dependent variable to establish a path model, to deeply and comprehensively explore the influencing factors of hospitalization cost for diabetic patients and their rank relationship.

Statistical analysis was performed using SPSS Statistics version 26 and Amos version 24 SPSS software. SPSS Statistics version 26 was mainly used for univariate analysis and the establishment of multiple linear regression models, while Amos software was used for the establishment of a path model. The statistical significance level of the above analysis was *p*-value < 0.05.

## 3. Results

### 3.1. The Results of Univariate Analysis of Length of Stay and Hospitalization Cost

As shown in Table 2, there were significant differences in the length of stay among diabetic patients of different genders, visit times, hospital levels, surgery and procedures, complications and comorbidities, and types of diabetes (*p* < 0.05), and there were significant differences in the length of stay among diabetic patients before and after DRG reform (*p* < 0.001). In addition, gender, visit times, length of stay, hospital level, surgery and procedures, complications and comorbidities, and type of diabetes had significant differences in the hospitalization cost for diabetic patients (*p* < 0.05).

### 3.2. The Results of Multivariate Analysis of Length of Stay and Hospitalization Cost

According to the results of multiple linear regression, the length of stay of diabetic patients was affected by the hospital level, whether accompanied by complications and comorbidities, whether accompanied by serious comorbidities and complications, whether the patients had type 2 diabetes mellitus, and whether the patients had other types of diabetes mellitus (*p* < 0.05), and the regression equation of length of stay (F = 25.803, *p* < 0.001, R^2^ = 0.156):

Y_1_ = 1.276 + 0.282 × X_4_ + 0.412 × X_6-1_ + 0.416 × X_6-2_ + 0.238 × X_7-1_ + 0.238 × X_7-2_ (1)

Hospitalization cost was affected by hospital level, whether accompanied by complications and comorbidities, whether accompanied by serious comorbidities and complications, whether the patients had other types of diabetes mellitus, the length of hospital stay (*p* < 0.05), and the regression equation of hospitalization cost (F = 484.023, *p* < 0.001, R^2^= 0.776):

Y_2_ = 4.527 + 0.792 × X_4_ + 0.245 × X_6-1_ + 0.131 × X_6-2_ + 0.110 × X_7-2_ + 0.676 × Y_1_ (2)

The specific results are shown in Table 3. According to the results of multiple linear regression, P_e_= $\sqrt{1-R^{2}}$= 0.473 of the residual model path coefficient of hospitalization cost could be calculated, which was lower than the standardized coefficient of Y_1_, indicating that there were other factors affecting hospitalization cost, and a path model could be established based on the results of multiple linear regression to comprehensively analyze the influencing factors of hospitalization cost.

### 3.3. Path Analysis Results of Hospitalization Cost

**Figure 1:**
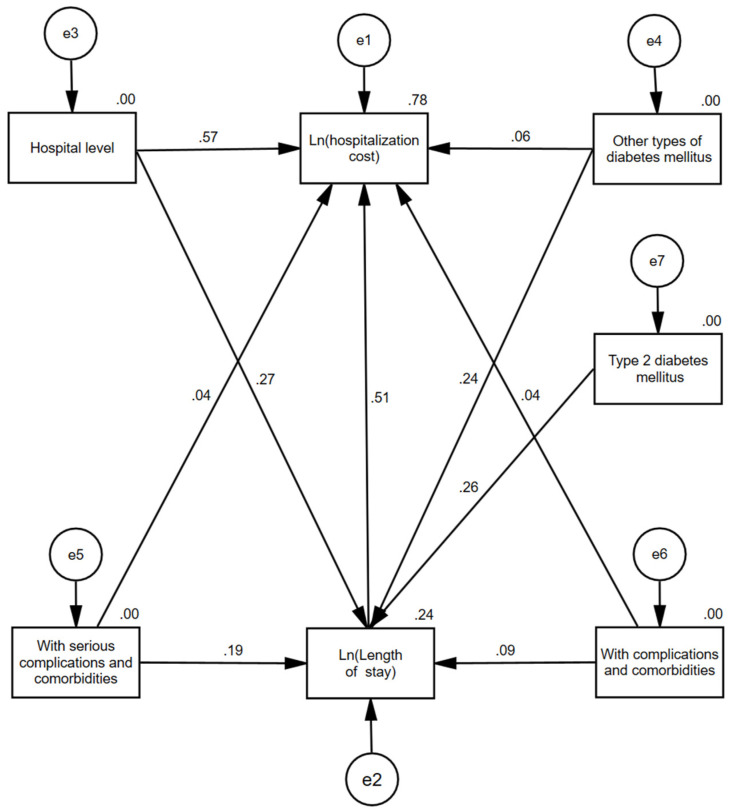
Path diagram of factors influencing hospitalization costs in diabetic patients.

As shown in Figure 1, hospital level, whether accompanied by minor complications and comorbidities, whether accompanied by serious complications and comorbidities, whether the patients had type 2 diabetes mellitus, and whether the patients had other types of diabetes mellitus all had indirect effects on hospitalization cost through the length of stay. In addition, hospital level, whether accompanied by minor complications and comorbidities, whether accompanied by serious complications and comorbidities, and whether the patients had other types of diabetes mellitus could also directly affect hospitalization cost.

According to the path model, the effect decomposition of the factors influencing hospitalization cost could be obtained, and the specific results were shown in Table 4. To clarify, the direct path coefficient of the independent variable to the dependent variable is equal to the standardized regression coefficient, the indirect path coefficient of the independent variable acting on the dependent variable Y_2_ through the intermediary variable Y_1_ is equal to the direct path coefficient of the independent variable to the intermediary variable Y_1_ × (the direct path coefficient of Y_1_ to Y_2_), and the total path coefficient is the sum of the direct path coefficient and the indirect path coefficient. Here, the path coefficient is the effect size. According to the above calculation and analysis method, the effects of various factors on hospitalization cost were ranked as follows: hospital level, length of stay, other types of diabetes mellitus, with serious complications and comorbidities, type 2 diabetes mellitus, and with complications and comorbidities.

## 4. Discussion

This study was one of the few subject designs that used TCM hospitals, which have strong Chinese medical and health characteristics, as the main focus for analysing the factors influencing hospitalization cost for diabetes patients. The research found that the length of stay and hospitalization cost for male diabetics staying in a TCM hospital were higher than for women, but the difference between the two was not very large. However, the length of stay and hospitalization cost of diabetic patients with a history of two or more visits were higher than those with one visit. More visit times meant that the disease was relatively complex, so the length of stay was longer and the medical cost was relatively higher. At the same time, the length of stay and hospitalization cost of diabetic patients in tertiary hospitals were significantly higher than those in secondary hospitals, and the major reason was that the Chinese government has been implementing a hierarchical diagnosis and treatment system, so that diabetic patients with complex conditions could be treated in hospitals with higher-level medical care, thus facing higher medical cost [13].

It was puzzling that patients with diabetes who had surgery and other medical procedures had lower hospital stays and lower hospital costs than those who did not have surgery and procedures. In the context of China’s health care policies, one explanation for this observation is that surgery and procedures involved a more formal medical service process, with excellent treatment outcomes, thereby reducing in-hospital time. In addition, surgery and procedures would obtain greater medical insurance reimbursement support, so the actual hospitalization cost was relatively low.

What was completely consistent with existing studies was that complications and comorbidities had a significant impact on the length of stay and hospitalization cost of hospitalized patients [14,15,16]. The more serious the complications and comorbidities of diabetic patients, the higher the length of stay and hospitalization cost. This was mainly because the more serious the complications and comorbidities were, the more complicated the patient’s condition was, and the more treatment time and cost were incurred, which was consistent with the results of a multi-country study [17]. In addition, patients with complications and comorbidities had longer length of stay and higher hospitalization cost and recurrence rates than those without complications and comorbidities [18,19,20]. 

Through this study, it was also found that patients with type 2 diabetes mellitus and other types of diabetes mellitus had longer length of stay and higher hospitalization cost compared to patients with type 1 diabetes mellitus, contrary to several studies in Europe [7,21,22], probably due to the different types of hospitals where the patients were studied and thus the different treatment protocols in treating the same type of disease, resulting in different cost outcomes. It is also evident from the results of the study analysis that the DRG health insurance reform policy currently being implemented nationwide by the Chinese government has led to some reduction in length of stay and hospitalization cost for diabetic patients in TCM hospitals, initially achieving its health insurance policy reform goals.

In conclusion, based on the analysis of the overall results, hospitalization cost for diabetic patients in Chinese medicine hospitals was affected by the hospital level, length of stay, the type of diabetes mellitus, and complications and comorbidities, and the hospital level had the greatest impact on the hospitalization cost. If the influence of the nature of the hospital on the hospitalization cost for diabetic patients was ignored, and only the influence of the diabetes patient’s situation on the hospitalization cost was considered, then the hospitalization cost of diabetic patients in a Chinese medicine hospital was mainly related to the length of stay, the type of diabetes, and the complications associated with comorbidities. In this case, the number of days in hospital was the strongest factor affecting hospitalization cost, which was consistent with the findings of Sima, D.I., Bala, C. et al. [23,24].

## 5. Research Deficiencies and Limitations

Firstly, this study only included diabetic patients from two TCM hospitals in Qingyang City, Gansu Province, China as the research focus, and the sample size was relatively small, which limited the study. Secondly, due to the different levels of economic development and uneven distribution of TCM resources in different provinces of China, the TCM hospitals in Qingyang City, Gansu Province, did not intuitively reflect the level of cost and treatment in TCM hospitals in China, so a multi-province sampling would have better results.

## 6. Conclusions

This study took 1268 diabetic patients from two DRG-reformed TCM hospitals in Qingyang City, Gansu Province, China as the research focus, and found that the main factors influencing hospitalization cost for diabetic patients in TCM hospitals were hospital level, length of stay, type of diabetes, and complications and comorbidities, etc., among which hospital level was the most important factor affecting hospitalization cost. It was also known from this analytical study that the higher the Chinese medicine hospital level, the longer the length of stay, the more serious the complications and comorbidities, the higher the hospitalization cost would be, and compared with type 2 diabetes mellitus and type 1 diabetes mellitus, other types of diabetes mellitus would bring a heavier economic burden to patients. Therefore, if China is to further effectively utilize the advantages of TCM in treating chronic diseases such as diabetes mellitus, it must further optimize the hierarchical medical treatment system and further improve the level of Chinese medicine in treating diabetes mellitus to alleviate and even effectively reduce the economic pressure that chronic diseases such as diabetes bring to the country and the people.

## Figures and Tables

**Table 1:** Variable assignment processing case.

Variable Attributes	Variable Codes	Variable Names	Dummy Variables	Variable Assignment
Endogenous variables	Y_1_	Length of stay ^1^	—	Ln (Length of stay)
	Y_2_	Hospitalization cost ^2^	—	Ln (Hospitalization cost)
Exogenous variables	X_1_	Gender	—	0 = Male, 1 = Female
	X_2-0_~X_2-2_	Age	<45 (Reference)	0, 0
			45~60	1, 0
			>60	0, 1
	X_3_	Visit times	—	0 = One time, 1 = Two times or more
	X_4_	Hospital level	—	0 = Secondary hospitals, 1 = Tertiary hospitals
	X_5_	Surgery and Procedures	—	0 = No, 1 = Yes
	X_6-0_~X_6-2_	Complications and comorbidities	With minor complications and complications (Reference)	0, 0
			With complications and comorbidities	1, 0
			With serious complications and comorbidities	0, 1
	X_7-0_~X_7-2_	Type of diabetes	Type 1 diabetes mellitus (Reference)	0, 0
			Type 2 diabetes mellitus	1, 0
			Other types of diabetes mellitus ^3^	0, 1
	X_8_	DRG reform ^4^	—	0 = Before the reform, 1 = After the reform

Note: ^1^ Length of stay: the amount of time that a diabetic patient is actually hospitalized for treatment, and the unit is “days”; ^2^ Hospitalization cost: actual medical cost for diabetic patients in hospital, and the unit is RMB “yuan”; ^3^ Other types of diabetes mellitus: “specific types of diabetes due to other causes” and “gestational diabetes mellitus” were included; ^4^ DRG reform: medical insurance payment reform based on diagnosis-related groups for control of rising healthcare cost, and the date for the reform was 1 October 2019.

**Table 2:** Univariate analysis results of the length of stay and hospitalization cost for diabetic patients.

Variable	Variable Categories	N (%)	Length of Stay		Hospitalization Cost	
			M (P_25_, P_75_) ^1^	Z/H-Value ^2^ /*p*-Value	M (P_25_, P_75_)	Z/H-Value /*p*-Value
Gender	Male	855 (67.43)	12 (9, 15)	−2.50/0.024	5314.50 (3603.10, 6566.02)	−3.369/0.001
	Female	413 (32.57)	11 (9, 14)		4757.46 (2421.91, 6366.14)	
Age	<45	196 (15.46)	12 (9, 15)	0.452/0.798	5129.18 (3531.65, 6176.28)	5.885/0.236
	45~60	526 (41.48)	11.5 (9, 15)		5013.27 (2881.32, 6560.91)	
	>60	546 (43.06)	12 (9, 15)		5301.25 (3189.56, 6744.10)	
Visit times	One time	1247 (98.34)	12 (9, 15)	−3.470/0.001	5173.08 (3060.79, 6518.52)	−2.856/0.004
	Two times or more	21 (1.66)	15 (12, 18)		6264.90 (5441.96, 7561.48)	
Length of stay	1~7	119 (9.38)	6 (4, 7)	951.037 /<0.001	2663.37 (1832.32, 3783.57)	520.768 /<0.001
	8~14	787 (62.07)	11 (9, 13)		4567.46 (2470.38, 5752.93)	
	15~21	305 (24.05)	16 (15, 18)		6879.41 (6028.12, 8088.07)	
	22~28	43 (3.39)	24 (23, 25)		8552.96 (7435.97, 10191.01)	
	29 and above	14 (1.10)	29.5 (29, 34)		10970.54 (9234.02, 14119.92)	
Hospital level	Secondary hospitals	250 (19.72)	9 (8, 10.25)	−13.509 /<0.001	2109.95 (1875.26, 2352.06)	−23.466 /<0.001
	Tertiary hospitals	1018 (80.28)	13 (10, 15)		5721.91 (4542.43, 7013.67)	
Surgery and Procedures	No	1258 (99.21)	12 (9, 15)	−2.503/0.012	5210.09 (3174.24, 6548.89)	−4.244/<0.001
	Yes	10 (0.79)	9 (8, 11.25)		2103.74 (1662.75, 2374.13)	
Complications and comorbidities	With minor complications and comorbidities	1210 (95.42)	11.5 (9, 15)	95.056/<0.001	5084.91 (3019.57, 6379.90)	78.581/<0.001
	With complications and comorbidities	11 (0.87)	19 (13, 21)		8485.96 (3264.42, 10576.09)	
	With serious complications and comorbidities	47 (3.71)	18 (17, 20)		8214.58 (6782.10, 9747.09)	
Type of diabetes	Type 1 diabetes mellitus	49 (3.86)	10 (7.5, 13.5)	7.895/0.019	4383.22 (3011.96, 5482.12)	7.673/0.022
	Type 2 diabetes mellitus	952 (75.08)	12 (9, 15)		5171.61 (3058.24, 6515.02)	
	Other types of Diabetes mellitus	267 (21.06)	12 (9, 15)		5510.98 (3553.35, 6826.97)	
DRG reform	Before the reform	831 (65.54)	12 (10, 15)	−3.492/<0.001	5261.07 (3874.11, 6518.28)	−1.804/0.071
	After the reform	437 (34.46)	11 (9, 14.5)		4967.36 (2150.00, 6562.42)	

Note: ^1^ M (P_25,_ P_75_): median (the first quartile, the third quartile); ^2^ Z/H-value: Mann–Whitney U test statistical value or Kruskal–Wallis H test statistical value.

**Table 3:** Multiple linear regression results in the length of stay and hospitalization cost of diabetic patients.

Variables	Ln (Length of Stay)				Ln (Hospitalization Cost)			
	B ^1^	Beta ^2^	t-Value	*p*-Value	B	Beta	t-Value	*p*-Value
Constant	1.276		10.014	<0.001	4.527		49.080	<0.001
Gender (ref = Male)								
Female	−0.008	−0.010	−0.366	0.714	−0.010	−0.008	−0.618	0.537
Visit times (ref = One time)								
Two times or more	0.145	0.048	1.842	0.068	−0.005	−0.001	−0.083	0.934
Hospital level (ref = Secondary hospitals)								
Tertiary hospitals	0.282	0.288	10.250	<0.001	0.792	0.585	40.543	<0.001
Surgery and Procedures (ref = No)								
Yes	0.010	0.002	0.084	0.933	0.009	0.002	0.111	0.911
Complications and comorbidities (ref = With minor complications and comorbidities)								
With complications and comorbidities	0.412	0.098	3.774	<0.001	0.245	0.042	3.128	0.002
With serious complications and comorbidities	0.416	0.202	7.702	<0.001	0.131	0.046	3.325	0.001
Type of diabetes (ref = Type 1 diabetes mellitus)								
Type 2 diabetes mellitus	0.238	0.264	4.480	<0.001	0.024	0.019	0.631	0.528
Other types of diabetes mellitus	0.238	0.249	4.146	<0.001	0.110	0.083	2.737	0.006
DRG reform (ref = Before the reform)								
After the reform	0.006	0.007	0.234	0.815				
Ln (Length of stay)					0.676	0.489	33.644	<0.001

Note: ^1^ B: unstandardized coefficients; ^2^ Beta: standardized coefficients.

**Table 4:** Effect decomposition table of influencing factors of hospitalization cost of diabetic patients.

Variables	Direct Effect	Indirect Effect	Total Effect	Ranking of Total Effect
Length of stay	0.509	--	0.509	2
Hospital level	0.573	0.139	0.713	1
Type 2 diabetes mellitus	--	0.130	0.130	5
Other types of diabetes mellitus	0.065	0.123	0.188	3
With complications and comorbidities	0.041	0.048	0.089	6
With serious complications and comorbidities	0.045	0.097	0.142	4

Note: The statistical significance for both the indirect and direct effects was *p* < 0.05, and “--” indicates there were no relevant effects.

## Data Availability

The study data should not be made public because they need to be treated confidentially, so please contact the corresponding author via email if necessary.
